# Use of bronchoscopic steam thermal ablation (BTVA) in a clinically compromised patient

**DOI:** 10.1186/s13019-022-01756-3

**Published:** 2022-02-07

**Authors:** Roberto Marchese, Chiara Lo Nigro, Federica Scaduto

**Affiliations:** Private Hospital La Maddalena: Casa di Cura La Maddalena SpA, Palermo, Italy

**Keywords:** COPD, BTVA, Bronchoscopy, Lung volume reduction, Emphysema

## Abstract

**Background:**

Bronchoscopic lung volume reduction (BLVR) techniques improve lung function and increase exercise tolerance in patients with chronic obstructive pulmonary disease (COPD) and BLVR treatment is included in the Global Initiative for Chronic Obstructive Lung Disease (GOLD) treatment guidelines for these patients. BTVA (Intervapor Uptake Medical, Tustin, CA, USA) represents a recent therapy of this group that allows to treat sublobar areas and for this reason is used clinically compromised patients, like in this case report.

**Case presentation:**

In this paper we describe a case report of an 85-year-old male with severe respiratory failure and a diagnosis of emphysema presented with dyspnea and clinical worsening, despite the best medical therapy practiced. For comorbidity and pathology’s features he was excluded from surgical treatment options, like lung volume reduction surgical (LVRS) and from positioning of endobronchial valves (EBV) for the presence of collateral ventilation and he was addressed to BTVA. The procedure was successful for this patient.

**Conclusions:**

This case supports recent suggestions that BTVA can be a good alternative treatment for patients properly selected.

## Background

Endoscopic lung volume reduction (ELVR), is included in the GOLD treatment guidelines for COPD patients [1] and represents an alternative approach to the LVRS. The endpoint of LVRS is reduce the hyperinflation to optimize the respiratory mechanics, leading to improved lung function and physical performance but is associated with high morbidity and mortality. ELVR has the same goal but lower risk. Several techniques of ELVR were developed in recent years, like valve therapy or coil implantation.

The most used devices are endobronchial valves (EBV) [2–4], used for the first time in 2002, but this treatment is reserved to patients with emphysema with no collateral ventilation (CV) and integrity of the interlobar fissures (IF) at the chest computed tomography (CT). For emphysematous patients without these features there are alternative devices [5, 6]. BTVA, bronchoscopic thermal vapor ablation, (Intervapor Uptake Medical, Tustin, CA, USA) represents a recent alternative therapy for these patients that uses high temperature water vapor to induce parenchymal thermal damage and inflammation resulting in fibrosis and volume reduction in the treated area, independently from interlobar CV [7–9].

## Case presentation

An 85-year-old male was admitted to our clinic (April 2019) with diagnosis of COPD (GOLD D). He had a history of arterial blood hypertension and hyperuricemia. A family history of lung tumor (brother), nor of drug allergies. He was a former smoker of 60 pack years (stop smoking 20 years prior). He had important dyspnea at rest (mMRC > 3) despite optimal pharmacotherapy (aclidinium bromide and salmeterolo/fluticasone) two times daily, teofilline, prednisone as needed and continuous 6 L/min of oxygen.

Pulmonary function test (PFT): ratio of forced expiratory volume in 1 s (FEV1) to forced vital capacity (FVC) was 51%, FEV1 was 1.29 L (percentage of predicted FEV1, 56%), residual volume (RV) was 3.96 L (percentage of predicted RV, 140%); the total lung capacity (TLC) was 7.50 L (percentage of predicted TLC, 118%), and the percentage of predicted diffusing capacity of carbon monoxide (DLCO) was 21%. Arterial blood gas analysis revealed a pH of 7.37, PaCO2 of 46.4 mmHg and PaO2 of 61.8 mmHg, SaO2 92.4% in room air. At 6MWT he walked greater than 140 mt (about 160 mt). St George Respiratory Questionnaire (SGRQ) was calculated. Echocardiography shows an ejection fraction of > 55%, with normal left and right ventricular size, normal PAPS.

**Fig. 1:**
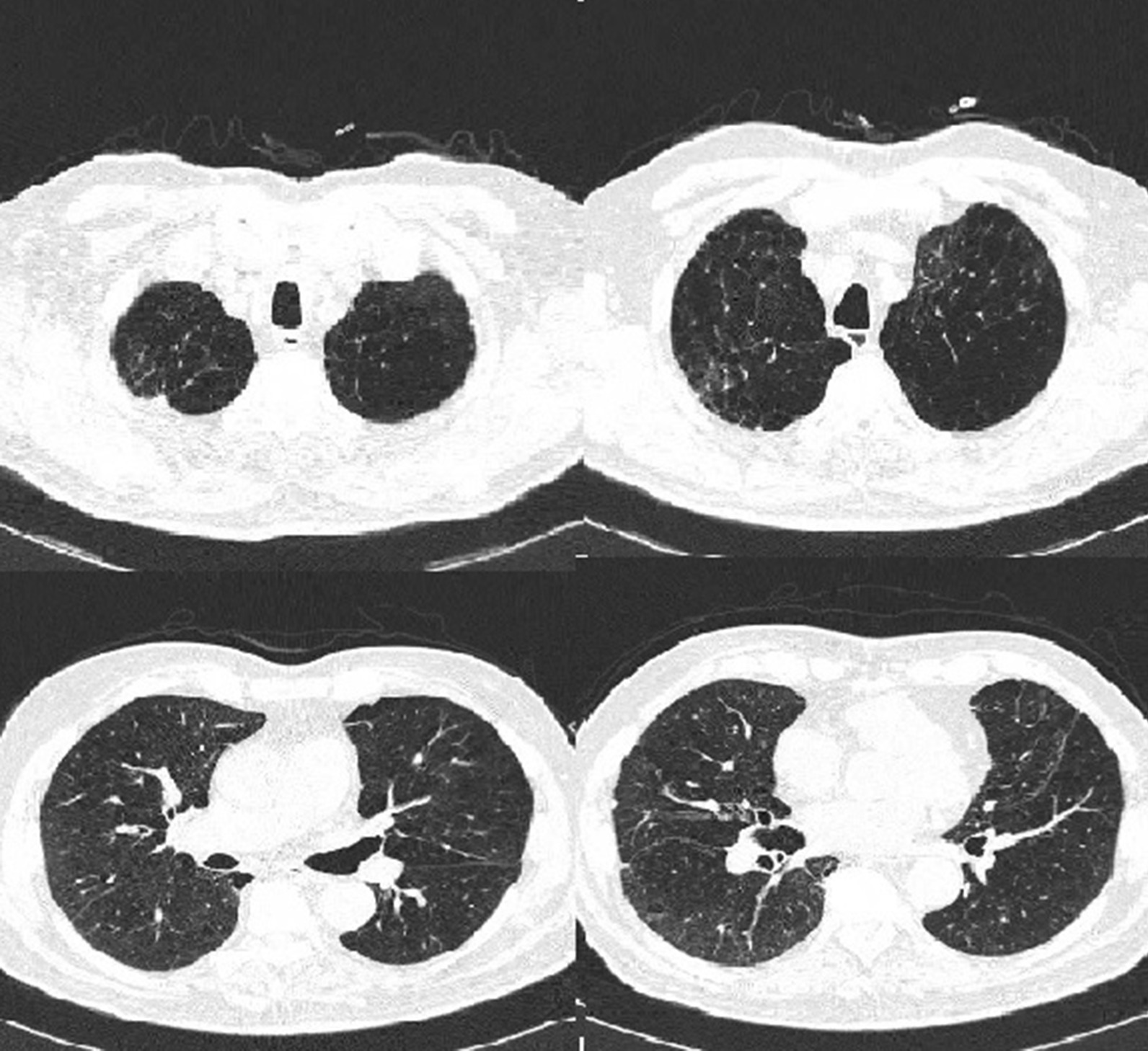
Chest tomography performed on July 2019: severe panlobular emphysema in both lungs with higher prevalence in upper lobes

CT performed in July 2019 indicated severe and heterogeneous pan-lobular emphysema with higher prevalence in upper lobes (UL) (Fig. 1).

So surgery was excluded cause of age, comorbidity and severe respiratory failure (6 L/min) and BTVA was preferred to EBV for the absence of IF and collateral ventilation.

In order to perform BLVR, the CT scan was analyzed by a dedicated software (VIDA Diagnostics, Coralville, IA, USA) measuring disease severity of each segment for identify the target area. The software showed a difference in lung density between targeted UL segment and its respective lower lobe and absence of IF. For this reason, LVRS and EBV weren’t taken into consideration and BTVA was proposed as an alternative.

The total procedure time was about 10 min. The treatment was performed in rigid bronchoscopy under general anesthesia, in order to suppress the cough that could displaced the balloon.

**Fig. 2:**
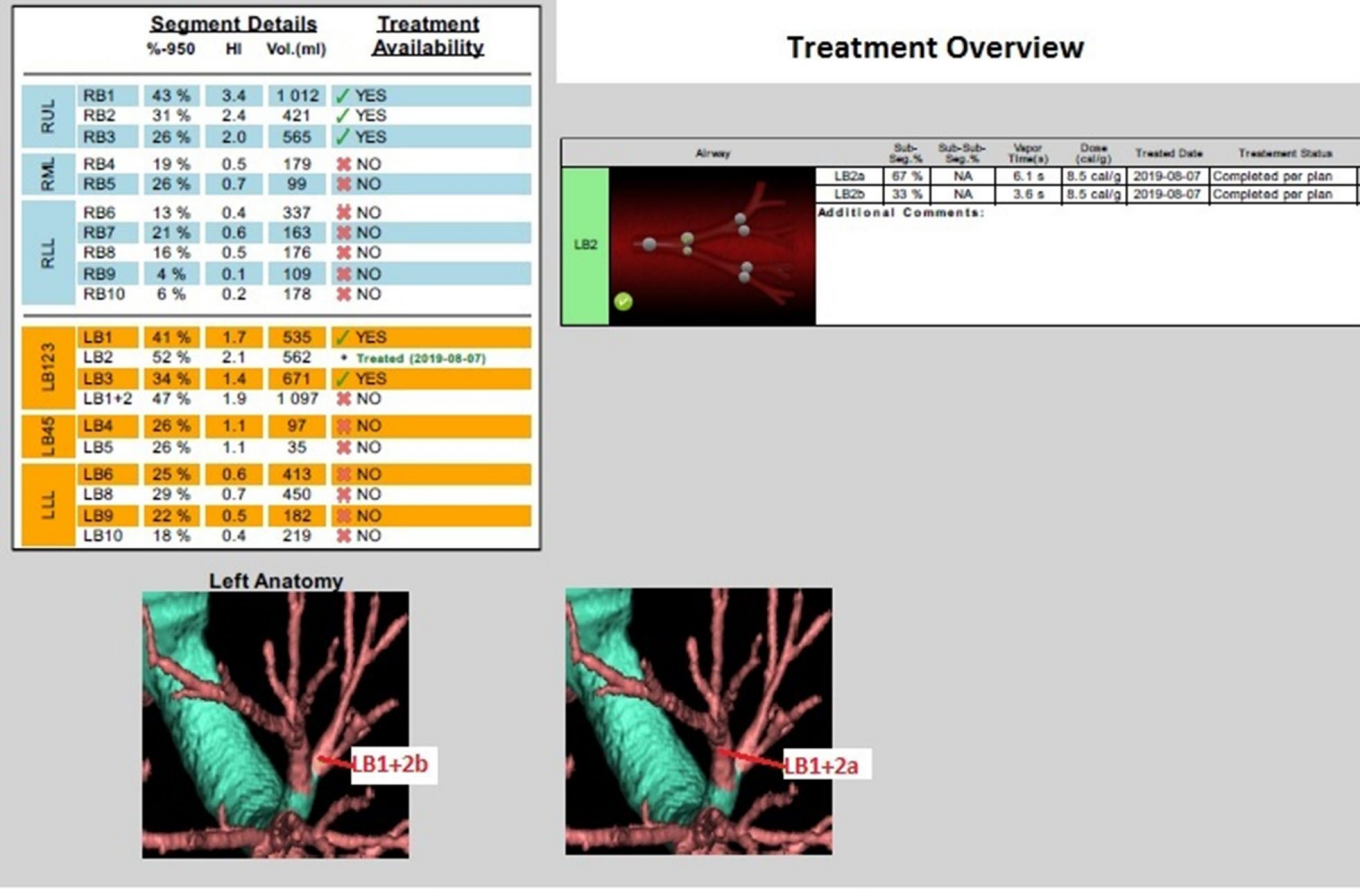
BTVA plan to treat LB 1 + 2 a-b. After placing an endoscopic catheter, a balloon was inflated in order to occlude the target segment and water heated vapor was delivered, 8.5 cal/g dose, first in LB1 + 2a for 6.1 s. Afterwards the balloon was deflated and the catheter was inserted into the other sub-segmentary bronchus LB1 + 2b for 3.6 s to complete the treatment of LB 1+ 2 a-b

After placing the catheter, a balloon was inflated in order to occlude the target segment and water heated vapor was delivered, 8.5 cal/g dose, first in LB1 + 2a for 6.1 s. Afterwards the balloon was deflated and the catheter was inserted into the other sub-segmentary bronchus LB1 + 2b for 3.6 s to complete the treatment of LB 1 + 2 a-b (Fig. 2).

After the procedure a transient increase in the inflammatory markers (PCR 5.48 mg/dL vs. 0.5 mg/dL at admission, PCT 0.13 µg/L vs. 0.02 µg/L) was found.

**Fig. 3:**
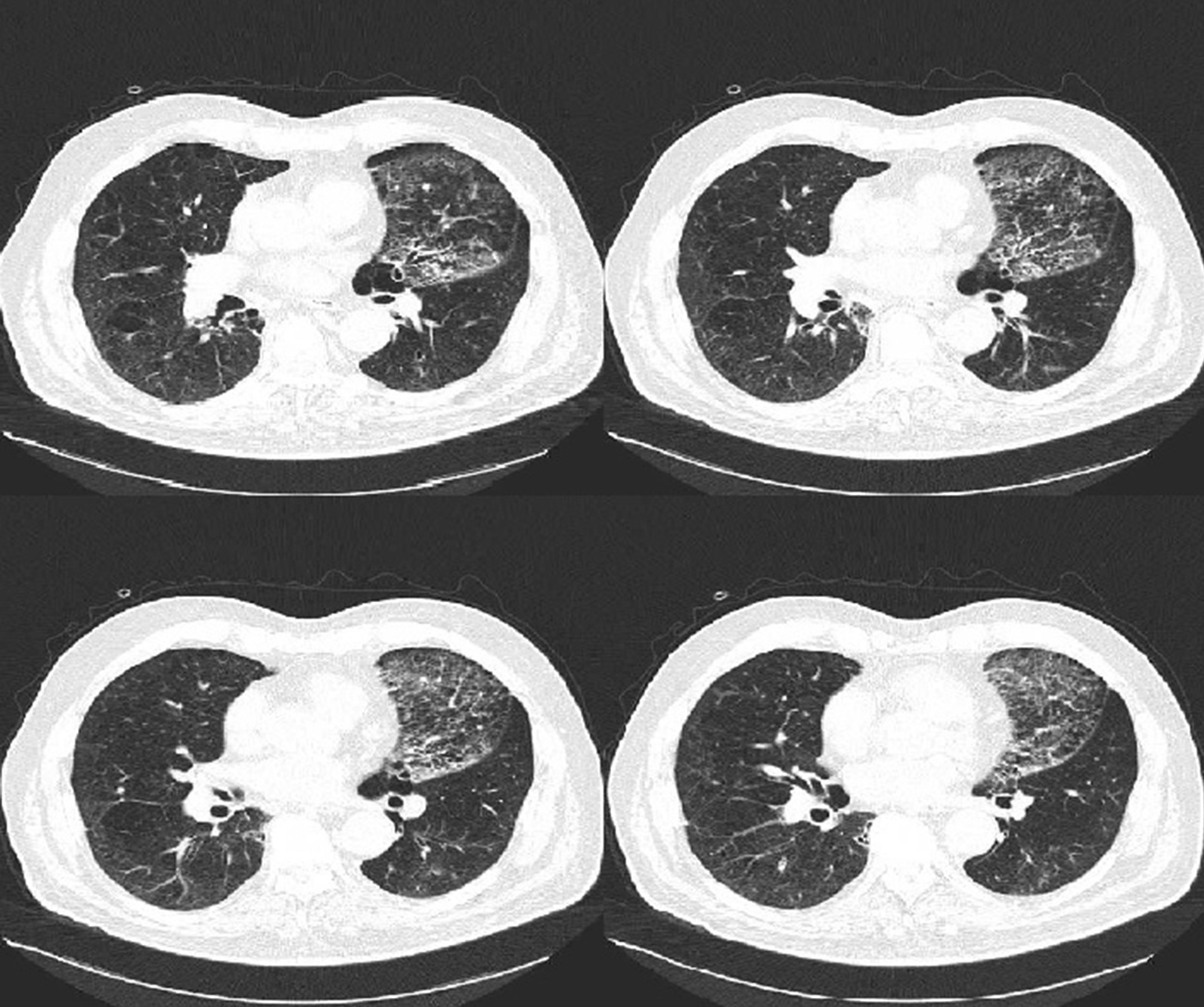
Chest tomography performed on August 2019, after the treatment. The CT scan shows a parenchymal pulmonary inflammatory response of the anterior segment of the left UL, with consequent reduction of lung volume

The CT scan performed after BTVA (Fig. 3) showed a parenchymal pulmonary inflammatory response of the apicodorsal segment of the left UL, with consequent reduction of lung volume [10]. No adverse events were recorded [11]. The patient was discharged at home in post operative day 8.

PFT was performed at 3 months after BTVA (October 2019). FEV1/FVC increased from 51 to 73%, FEV1 increased by 560 mL from 1.29 L (percentage of predicted FEV 56%) to 1.86 L (82%), RV decreased from 3.96 L (percentage of predicted RV, 140%) to 3.22 L (113%), TLC decreased from 7.50 L (percentage of predicted TLC, 118%) to 6.76 L (107%). The symptoms and quality of life improved markedly (SGRQ-C ≤  − 8 points). The oxygen therapy was reduced to 1 L/min.

During the follow-up (November 2019) he performed CT scan that identified a subpleural lung nodule in the posterior segment of the RUL (SUV 2.3).

On January 2019 a lung biopsy-CT guided was performed. The histological examination showed a carcinoma (CK7+, TTF1-, P40-) stage IIB (pT3 cN0 cM0). The patient was candidate to thermoablation because of the concomitant pathologies.

## Discussion and conclusion

BTVA represents a valid alternative to EBV that allows to treat only the most damaged lung segments of patients with heterogeneous emphysema. In particular, our patient for his age and for the presence of various comorbidity (COPD in oxygen therapy for cronic respiratory failure, previous hemorrhagic gastroduodenitis, frequent bronchial exacerbations with two hospitalization for bronchopneumonia) is not candidate to reduction volume surgery.

Infact among selection criteria for better results in LVRS, according to NETT Trial, there are the age < 70, FEV1 value between 20 and 40%, pCO2 value less than 45% and finally RV > 300%, characteristics that our patient doesn’t possess at the time of selection. LVRS also exclude from treatment patient with recurrent bronchopulmonary infections.

Furthermore with this technique we had the possibility to treat a patient with RV < 180% (in particular the patient has RV 140%), while through endobronchial valve treatment exist a cut-off of 180% and it allows to treat patient with collateral ventilation that is very common in patient with emphysema. The treatment was efficacy and the patient is still alive.

In 2009, Snell et al. [12, 13] proposed the use of BTVA. STEP-UP is the most important randomized, controlled open label trial [14, 15], that shows significant difference in FEV1 and SGRQ at 6 and 12 months [16]. In this trial patient have a mean FEV1 of 33% and a mean VR of 235% and undergone to a first treatment of 1 emphysematous segment of an upper lobe and then of one emphysematous segments in the contralateral upper lobe after 12 weeks. After 6 months there were statistically significant difference in FEV1 and SGRQ. Data obtained at the 12-month follow-up visits showed persistent improvements in lung function relative to baseline.

While Gompelmann et al. [17, 18] have emphasized that patients should have 20% > FEV1 < 45% of predicted values, RV > 175% of predicted values and DLCO ≥ 20% of predicted values and the patient had higher FEV1 and lower RV values, the outcome of the procedure was excellent.

As helium dilution technique was used to perform PFT, it’s possible that RV values are underestimated compared to the same values calculated with plethysmography. This suggest that is important use the latter technique to perform PFT.

Despite we treat only sub-segmentary bronchus LB 1 +  2a-b, because of the patient critical issue, an extraordinary result has been achieved.

It’s a procedure available in a great number of patients which are excluded from surgery or EBV for clinical and technical issues; it’s well tolerated and seems to be correlated with an improvement of quality of life. As the procedure depends on irreversible inflammatory reaction, patient selection is essential.

## Data Availability

Data sharing is not applicable to this article as no datasets were generated or analysed during the current study.

## Abbreviations

BLVR: Bronchoscopic lung volume reduction
COPD: Chronic obstructive pulmonary disease
GOLD: Global Initiative for Chronic Obstructive Lung Disease
LVRS: Lung volume reduction surgical
EBV: Endobronchial valves
BTVA: Bronchoscopic steam thermal ablation
CV: Collateral ventilation
IF: Integrity of the interlobar fissures
CT: Chest computed tomography
mMRC: Modified British Medical Research Council Questionnaire
PFT: Pulmonary function test
FEV1: Forced expiratory volume in 1 s
FVC: Forced vital capacity
RV: Residual volume
TLC: The total lung capacity
DLCO: Diffusing capacity of carbon monoxide
6MWT: Six minute walking test
SGRQ: St George Respiratory Questionnaire
PAPS: Pulmonary artery systolic pression
UL: Upper lobes
LB: Left bronchus

## References

[1] Neumeier A, Keith R (2020). Clinical guideline highlights for the hospitalist: the GOLD and NICE guidelines for the management of COPD. J Hosp Med.

[2] Davey C, Zoumot Z, Jordan S (2015). Bronchoscopiclung volume reduction with endobronchial valves for patients with heterogeneous emphysema and intact interlobar fissures (the BeLieVeR-HIFi study): a randomised controlled trial. Lancet.

[3] Klooster K, ten Hacken NH, Hartman JE, Kerstjens HA, van Rikxoort EM, Slebos DJ (2015). Endobronchial valves for emphysema without interlobar collateral ventilation. N Engl J Med.

[4] Koster TD, Slebos DJ (2016). The fissure: interlobar collateral ventilation and implications for endoscopic therapy in emphysema. Int J Chron Obstruct Pulmon Dis.

[5] Gompelmann D, Eberhardt R, Herth FJ (2015). Novel endoscopic approaches to treating chronic obstructive pulmonary disease and emphysema. Semin Respir Crit Care Med.

[6] Xu W, Wang J, He X (2020). Bronchoscopic lung volume reduction procedures for emphysema: a network meta-analysis. Medicine (Baltimore).

[7] Bandyopadhyay S, Henne E, Gupta A (2015). Segmental approach to lung volume reduction therapy for emphysema patients. Respiration.

[8] Herth FJ, Ernst A, Baker KM (2012). Characterization of outcomes 1 year after endoscopic thermal vapor ablation for patients with heterogeneous emphysema. Int J Chron Obstruct Pulmon Dis.

[9] Pu J, Wang Z, Gu S (2014). Pulmonary fissure integrity and collateral ventilation in COPD patients. PLoS ONE.

[10] Gompelmann D, Eberhardt R, Ernst A (2013). The localized inflammatory response to bronchoscopic thermal vapor ablation. Respiration.

[11] Emery MJ, Eveland RL, Eveland K (2010). Lung volume reduction by bronchoscopic administration of steam. Am J Respir Crit Care Med.

[12] Snell GI, Hopkins P, Westall G (2009). A feasibility and safety study of bronchoscopic thermal vapor ablation: a novel emphysema therapy. Ann Thorac Surg.

[13] Snell G, Herth FJ, Hopkins P (2012). Bronchoscopic thermal vapour ablation therapy in the management of heterogeneous emphysema. Eur Respir J.

[14] Valipour A, Herth FJ, Eberhardt R (2014). Design of the randomized, controlled sequential staged treatment of emphysema with upper lobe predominance (STEP-UP) study. BMC Pulm Med.

[15] Herth FJ, Valipour A, Shah PL (2016). Segmental volume reduction using thermal vapour ablation in patients with severe emphysema: 6-month results of the multicentre, parallel-group, open-label, randomised controlled STEP-UP trial. Lancet Respir Med.

[16] Shah PL, Gompelmann D, Valipour A (2016). Thermal vapour ablation to reduce segmental volume in patients with severe emphysema: STEP-UP 12 month results. Lancet Respir Med.

[17] Gompelmann D, Shah PL, Valipour A (2018). Bronchoscopic thermal vapor ablation: best practice recommendations from an expert panel on endoscopic lung volume reduction. Respiration.

[18] Gompelmann D, Eberhardt R, Herth FJ (2014). Technology update: bronchoscopic thermal vapor ablation for managing severe emphysema. Med Devices (Auckl).

